# Source identification and toxicity apportionment of polycyclic aromatic hydrocarbons in surface soils in Beijing and Tianjin using a PMF-TEQ method

**DOI:** 10.1371/journal.pone.0268615

**Published:** 2022-06-30

**Authors:** Huashuang Zhang, Qi Huang, Ping Han, Zhicheng Zhang, Shengtao Jiang, Wei Yang

**Affiliations:** 1 Institute for Brain Research and Rehabilitation, South China Normal University, Guangzhou, Guangdong, China; 2 College of Life Science, Taizhou University, Taizhou, Zhejiang, China; 3 Shandong Urban Construction Vocational College, Jinan, Shandong, China; 4 Polar and Marine Research Institute, Jimei University, Xiamen, Fujian, China; Universidade do Porto Centro Interdisciplinar de Investigacao Marinha e Ambiental, PORTUGAL

## Abstract

Beijing and Tianjin are two of the largest cities in northern China with high population densities and highly developed manufacturing industries. In the past decade, some authors have reported their PAH concentrations in surface soils, identified their sources and quantitatively reported their health risks. However, the contributions of different PAH sources to their toxicity have not been reported thus far. In this study, we reviewed the PAH concentrations, contributions of different sources to the toxicity, and cancer risks in soils from different land use types found within Beijing and Tianjin from data gathered by 41 studies. The total PAH concentration varied in the range of 175.7–1989.0 ng g^-1^ with a higher median PAH concentration detected in urban soils (789.7 ng g^-1^), followed by suburban soils (647.3 ng g^-1^) and rural soils (390.8 ng g^-1^). Source identification using diagnostic ratios and principal component analysis (PCA) suggested that the PAHs in all three land use types mainly originated from biomass and coal combustion, vehicular emissions, and petrogenic processes with contributions varying from 13% to 62%. Furthermore, results from a positive matrix factorization (PMF) model suggested that vehicular emissions and coal combustion in urban soils, and the vehicular emissions, coal combustion and biomass combustion in suburban and rural soils dominated the total PAH concentrations (>85%). These results were consistent with those of the PCA model. Results of the additional toxicity apportionment performed using the PMF model suggested that vehicular emissions and coal combustion contributed the most to the toxic equivalent quantity for Benzo(a)Pyrene (BaP_TEQ_) and, by extension, to the carcinogenic potencies. The incremental lifetime cancer risk (ILCR) values suggested a low risk level for adults exposed to PAHs in the different land use types found within Beijing and Tianjin.

## 1 Introduction

Polycyclic aromatic hydrocarbons (PAHs) are a group of pollutants with highly stable chemical structures that can be originated by different sources [1–3]. Although natural sources such as the petrogenic process can generate PAHs, anthropogenic sources, such as the incomplete combustion of biomass, coal, diesel, and other fossil fuels, the direct release of oil and its products, and their emissions from power generation plants and coke ovens, contribute more to their concentrations in the natural environment [4–6]. Once released into the environment, PAHs are transported and distributed within different environmental media, such as soils and sediments, water bodies, and the atmosphere. Surface soils are considered to be large reservoirs and sinks for PAHs, due to the physicochemical properties of soils that enable the adsorption of PAH compounds to soil particles with high organic matter content [3,7,8].

Various studies, including work done by the International Agency for Research on Cancer (IARC), have shown that PAHs are a group of toxic compounds with ecotoxic, genotoxic, mutagenic, and estrogenic effects [5–7,9]. Additionally, their adverse effects intensify with their accumulation and amplification in biological organisms [2,5,7]. In order to evaluate the cancer risks posed by the PAHs in soils and other environmental media, a health risk assessment model and an incremental lifetime cancer risk (ILCR) assessment model were proposed by the US Environmental Protection Agency (US EPA), two approaches which have been widely used by various authors [8,10,11]. Considering the different carcinogenic potencies of different PAH compounds, the toxic equivalent quantity for Benzo(a)Pyrene (BaP_TEQ_) calculated from the PAH concentrations and their toxic equivalent factors (TEFs) were used in the cancer risk assessment [1,6,7]. However, the contributions of different PAH sources to the BaP_TEQ_ concentration and their associated cancer risks were rarely reported in the literature.

In order to apportion the BaP_TEQ_ concentration and cancer risks to different PAH sources, the potential PAH sources should first be identified, and their source contributions subsequently calculated using a different method. Finally, the contributions of different PAH sources to the BaP_TEQ_ concentration and cancer risks were obtained. In source identification, although diagnostic ratios were used by some authors, it cannot provide accurate information regarding the PAH sources [12–15]. The principal component analysis (PCA) model can attribute portions of the total PAH concentration to different sources, but cannot apportion the BaP_TEQ_ concentration and cancer risks to each source [8,16]. Studies suggested that the chemical mass balance (CMB) model and positive matrix factorization (PMF) model could apportion the PAH concentrations in every sampling station to different PAH sources [17–20]. One study [18] used a CMB model to identify the PAH sources which contributed to the BaP_TEQ_ concentrations in the Liao River estuary wetland. However, the apportionment of BaP_TEQ_ concentrations and cancer risks to each source using the PMF model was rarely reported in the literature.

Beijing and Tianjin are two of the largest cities in northern China and have high population densities [13,14,21,22]. Highly developed manufacturing industries, including the oil exploration and refining, coke production, and the domestic coal combustion and biomass burning are the main PAH sources in this area, which likely adversely affect human health [13,21–24]. In the past decade, some authors have reported the PAH concentrations in surface soils in Beijing [19,21,23] and Tianjin [13,14,22], identified their sources using different methods [16,21,23,25], and quantitatively reported the health risks associated with exposure to PAHs in surface soils [19,23,25]. However, the contributions of different PAH sources to the BaP_TEQ_ concentration have not been reported thus far.

In this study, we intended to investigate the PAH pollution in different medias, for example the air and surface soil in Beijing and Tianjin. Unfortunately, there is no adequate PAH data in air samples, and the significant seasonal and spatio-temporal variability that controlled by the air temperature, wind directions and wind velocity would make the result suspectable. Thus, this study only discussed the PAH pollution in surface soil. We firstly investigated the PAH concentrations in different land use types (urban, suburban, and rural soils) within Beijing and Tianjin, and identified their emission sources using different methods. Subsequently, we apportioned the BaP_TEQ_ concentration to each PAH source with the PMF model. Finally, we quantitatively evaluated their cancer risks using an ILCR model.

## 2 Material and methods

### 2.1 Data collection and quality control

After the 2008 Olympic Games, the Beijing government has made great efforts to manage the environmental pollution, such as relocating many large refineries, controlling the number of fuel vehicles and optimizing the energy structure. Study has shown that the environmental pollution in Beijing is improving. Here, we want to assess the PAH pollution in surface soil in Beijing and Tianjin science 2010 after the Beijing Olympic Games.

In this study, the concentrations of 16 US priority control PAHs, including naphthalene (Naph, 2-ring), acenaphthene (Ace, 3-ring), acenaphthylene (Acy, 3-ring), phenanthrene (Phe, 3-ring), fluorene (Flu, 3-ring), anthracene (Ant, 3-ring), pyrene (Pyr, 4-ring), fluoranthene (Flt, 3-ring), benz[a]anthracene (BaA, 5-ring), chrysene (Chr, 4-ring), dibenzo[a,h]anthracene (DBahA, 5-ring), benzo[a]pyrene (BaP, 5-ring), benzo[b]fluoranthene (BbF, 5-ring), benzo[k]fluoranthene (BkF, 5-ring), benzo[g,h,i]perylene (BghiP, 6-ring), and indeno[1,2,3-cd]pyrene (IND, 6-ring), in surface soils from Beijing and Tianjin were obtained from peer-reviewed literatures published from 2010 to 2021 on various websites (including the Springer, Google Scholar, ScienceDirect, Wiley, China National Knowledge Internet [CNKI] and Web of Knowledge). Different search terms (for example “Polycyclic Aromatic Hydrocarbons,” “PAHs,” “Beijing,” “Tianjin,” “Soil,” and “China”) were used in retrieving the relevant papers. Only those papers that listed the concentrations of all 16 PAH compounds were selected. Three additional eligibility criteria that were necessary for the inclusion of a study in the analysis were: 1) surface soils were collected at the depth of 0–20 cm; 2) soil samples were collected from core urban areas (including parks, universities, schools, roadsides with heavy or light traffic, and residential, plantation, and vacant areas), suburban and rural areas (including the agricultural and residential areas); 3) the PAH analyses were conducted with strict quality control measures including the use of laboratory blanks, matrix-spiked recoveries, duplicates, and certified reference PAHs. In this study, the PAH concentrations in contaminated sites with intensive industrial activities were not included owing to their extremely high PAH concentrations with definite, known emission sources, such as the coking plant [26], iron and steel industrial site [27] and petroleum-contaminated area [28].

A total of 41 cases from >100 studies met the criteria, and the PAH concentrations and sampling depths in different study areas are summarized in Table 1. The detailed information concerning analytical procedures is presented in S1 Table, including instrumentation, recoveries, quality control and assurance, and the method detection limit (MDL).

**Table 1:** Summary of PAHs concentrations in different land use types in Beijing and Tianjin.

Land use types	Soil descriptions	Total PAHs (ng g^-1^)	BaPeq (ng g^-1^)	Sampling depth (cm)	Contamination levels	Reference
Urban Soils	Surface soil in urban parks in Beijing	460.0	50.7	0–10	Weakly contaminated	[21]
	Urban soils in Beijing	1228.0	159.2	0–10	Heavily contaminated	[25]
	Surface soils in Nankai University, Tianjin	360.0	36.9	0–20	Weakly contaminated	[29]
	Surface soils in Beijing	1082.6	180.7	0–20	Heavily contaminated	[16]
	Surface soil in schools in Beijing	1989.0	286.6	0–10	Heavily contaminated	[30]
	Surface soil in parks in Beijing	1285.0	170.6	0–10	Heavily contaminated	[30]
	Surface soil in roadside with heavy traffic in Beijing	1026.0	135.0	0–10	Heavily contaminated	[30]
	Surface soil in residential area in Beijing	811.0	98.7	0–10	Contaminated	[30]
	Surface soil in plantation area in Beijing	673.0	85.3	0–10	Contaminated	[30]
	Surface soil in roadside with light traffic in Beijing	538.0	68.9	0–10	Weakly contaminated	[30]
	Surface soil in vacant area in Beijing	523.0	65.4	0–10	Weakly contaminated	[30]
Suburban Soils	Surface soils in Xiqing, Tianjin	1490.0	178.7	0–20	Heavily contaminated	[29]
	Surface soils in Jinnan, Tianjin	708.0	85.4	0–20	Contaminated	[29]
	Surface soils in Beicheng, Tianjin	904.0	69.6	0–20	Contaminated	[29]
	Surface soils in Dongli, Tianjin	699.0	48.8	0–20	Contaminated	[29]
	Surface soils in Jinghai, Tianjin	142.0	12.9	0–20	Not contaminated	[29]
	Surface soils in Jixian, Tianjin	382.0	32.7	0–20	Weakly contaminated	[29]
	Surface soils in Xiqing, Tianjing	422.8	57.1	0–20	Weakly contaminated	[31]
	Surface soils from Tianjin coastal new region	932.0	124.2	0–10	Contaminated	[22]
	Surface soil in Tongzhou District, Beijing	1004.1	158.4	0–20	Heavily contaminated	[32]
	Surface soils in suburban area of Beijing	321.8	38.1	--	Weakly contaminated	[23]
	Surface soils in suburban area of Beijing and Tianjin	622.4	54.6	0–5	Contaminated	[14]
Rural Soils	Agricultural soil in suburb of Beijing	460.8	24.8	0–20	Weakly contaminated	[33]
	Surface soils in rural area of Beijing	219.2	27.3	--	Weakly contaminated	[23]
	Surface soils in rural area of Beijing and Tianjin	195.3	14.8	0–5	Not contaminated	[14]
	Arable soils of Beijing	489.6	71.0	0–10	Weakly contaminated	[19]
	Agricultural soil in Tianjin	1295.8	185.6	0–5	Heavily contaminated	[12]
	Surface soil from garden in Tianjin	1258.6	126.8	0–20	Heavily contaminated	[13]
	Surface soil from cropland in Tianjin	624.7	114.4	0–20	Contaminated	[13]
	Surface soil from dryland in Tianjin	1003.9	97.1	0–20	Heavily contaminated	[13]
	Surface soil in residential areas of Tianjin	481.8	4.5	0–20	Weakly contaminated	[34]
	Surface soil in residential areas of Tianjin	435.1	10.8	0–20	Weakly contaminated	[34]
	Surface soil in residential areas of Tianjin	289.1	3.7	0–20	Weakly contaminated	[34]
	Surface soil in agricultural facility areas of Tianjin	175.7	6.9	0–20	Not contaminated	[34]
	Surface soil in agricultural facility areas of Tianjin	296.1	25.5	0–20	Weakly contaminated	[34]
	Surface soil in agricultural facility areas of Tianjin	229.3	11.9	0–20	Weakly contaminated	[34]
	Surface soil in agricultural facility areas of Tianjin	286.0	17.0	0–20	Weakly contaminated	[34]
	Surface soil in farmland around livestock breeding areas of Tianjin	772.9	10.9	0–20	Contaminated	[34]
	Surface soil in farmland around livestock breeding areas of Tianjin	259.9	4.1	0–20	Weakly contaminated	[34]
	Surface soil in farmland around industrial areas of Tianjin	323.3	55.2	0–20	Weakly contaminated	[34]
	Vegetable soils from the Beijing-Tianjin	602.5	111.4	0–20	Contaminated	[24]

In order to describe their concentrates, distribution patterns, potential sources, and health risks more conveniently in the following discussion, three different land use types, urban (11 studies), suburban (11 studies), and rural soils (19 studies) in Beijing and Tianjin were considered in this study.

### 2.2 Source identification and toxicity apportionment

#### 2.2.1 Diagnostic Ratios and PCA

PAHs formed under different combustion conditions have different diagnostic ratios, which can be an effective method to identify the potential sources of PAHs [19,22,35]. Examples of diagnostic ratios of PAHs which are applied to identify their possible emission sources include BaA/228, Flt/202, Flt/(Flt+Pyr), Ant/(Ant+Phe), BaA/(BaA+Chr), Ant/(Ant+Phe), and IND/(IND+BghiP) [12–15]. In this study, we used the diagnostic ratios of Flt/(Flt+Pyr), Ant/(Ant+Phe), and BaA/(BaA+Chr) to obtain information regarding their sources in the following discussion. Generally, the ratio of BaA/(BaA+Chr) was used to distinguish petroleum emissions from those of wood and coal combustion. Values higher than 0.35 are typically associated with wood and coal combustion, whereas ratio values lower than 0.2 indicate a petroleum source. The ratios varied in the range of 0.2–0.35, which suggests a mixed source of petroleum and combustion [3,15,36]. It was reported that a ratio value of Flt/(Flt+Pyr) that is lower than 0.4 indicates a petroleum source, whereas ratios between 0.4 and 0.5 indicate petroleum combustion, and a ratio greater than 0.5 is indicative of biomass and coal combustion [7,37–39]. Prior studies suggested that a ratio of Ant/(Ant + Phe) that is less than 0.1 implies a petrogenic source, and ratios greater than 0.1 indicate a petroleum combustion source [15,39].

As criticized by several authors for their known uncertainties, diagnostic ratios cannot definitively identify the emission sources [26,27,35]. In most cases, additional information concerning the specific PAH sources is necessary for their controls, and a PCA model is usually used as a supplementary technique to identify their emission sources [8,26,27]. The PCA model is a method that extracts valuable information from multivariate datasets. Using the orthogonal transformation method, two or three principal components (PCs) with eigenvalues >1.0 were extracted [16,19]; subsequently, based on the different factor loadings, the potential PAH sources for each PC were evaluated and identified by the source markers or profiles [8,16]. Finally, in order to assess the contribution of each identified source to the total PAH concentrations quantitatively, a multiple linear regression (MLR) model was also produced [16].

#### 2.2.2 Source identification with a PMF model

A PMF model developed by the USEPA was also used in the PAH source identification in this study [6,17,19,21]. A brief introduction of the PMF model is presented as follows:

First, it defines a n×m data original matrix X, which could be factorized into two matrices (G (n×p) and F (p×m)) with an unexplained part E (n×m), as:

X=G⋅F+E (1)

where n and m represent the number of samples and chemical species, respectively [19,21]. Thus, the concentration of the j^th^ chemical species measured in the i^th^ sample (*x*_ij_) was expressed as:

$$x_{ij}=\sum_{k=1}^{p}g_{ik}f_{kj}+e_{ij}$$ (2)

where g_ik_ and f_kj_ were the contribution of source k to the i^th^ sample and the concentration of the j^th^ chemical species in source k, respectively. e_ij_ was the residual item in the calculation.

The aim of the PMF model is to minimize the objective function Q related to e_ij_ and uncertainty (u_ij_) for deriving source contributions and profiles:

$$Q=\sum_{i=1}^{n}\sum_{j=1}^{m}{\left(\frac{e_{ij}}{u_{ij}}\right)}^{2}$$ (3)

where e_ij_ is the difference between the observations and the modeled values, and u_ij_ is the uncertainty in the x_ij_ measurement and is related to the MDL of each species and the species-specific error fraction [19,22,39].

Two types of uncertainty, i.e., sample-specified and equation-based, were provided in the PMF model. The equation-based uncertainty (U_nc_) was adopted in this study and was calculated using the following equations:

$$U_{\mathrm{nc}}=\frac{5}{6}\times MDL\;(\mathrm{If}\;\mathrm{concentration}≦\mathrm{MDL})$$ (4)

$$U_{\mathrm{nc}}=\sqrt{{\left(\mathrm{Error}\;\mathrm{Fraction}\times Concentration\right)}^{2}+MDL^{2}}\;(\mathrm{If}\;\mathrm{concentration}>\mathrm{MDL})$$ (5)

where Error Fraction is the percentage uncertainty in the determination of the variable, and is normally estimated as the standard deviations of deuterated surrogate recoveries [19,21].

#### 2.2.3 Toxicity apportionment using a PMF-TEQ method

In this study, the carcinogenic potencies for the 16 PAH compounds were evaluated based on their toxic equivalent factors (TEFs), which were expressed as BaP_TEQ_. The BaP_TEQ_ concentrations were calculated by multiplying the individual PAH concentrations and their TEF values as follows [1,6,7]:

$$B{\mathrm{aP}}_{TEQ}=\sum_{i=1}^{16}\left(TEF_{i}\times (PAH_{i}\;Concentration)\right)$$ (6)

where the TEF_i_ is the toxic equivalent factor for a specific PAH compound i, as shown in Table 2 [19,22,40].

**Table 2:** TEFs used in calculating the carcinogenic potency.

PAHs	TEFs^a^	PAHs	TEFs^a^
Naph	0.001	BaA	0.1
Acy	0.001	Chr	0.01
Ace	0.001	BbF	0.1
Flu	0.001	BkF	0.1
Phe	0.001	BaP	1
Ant	0.01	IND	0.1
Flt	0.001	DBahA	1
Pyr	0.001	BghiP	0.01

^a^ Adopted from Nisbet and LaGoy [40].

Based on the results of the PMF model, the contribution of each PAH source to their toxicity was quantitatively estimated followed a method described in [18,20] and [41] with the following equations:

$${\left(B{\mathrm{aP}}_{TEQ}\right)}_{\mathrm{kp}}=\sum_{i=1}^{16}\left(TEF_{i}\times {\left(PAH_{i}\right)}_{\mathrm{kp}}\right)$$ (7)

$${\left(PAH_{i}\right)}_{\mathrm{kp}}=S_{kp}\times f_{ip}$$ (8)

where (BaP_TEQ_)_kp_ is the calculated contribution of the p^th^ source to BaP_TEQ_ in the k^th^ soil sample, (PAH_i_)_kp_ is the estimated contribution of the p^th^ source for i^th^ PAH species in the k^th^ soil sample, S_kp_ is the contribution of the p^th^ source in the k^th^ soil sample, which was obtained from the PMF model; f_ip_ is the fraction of i^th^ PAH species in p^th^ source profile.

### 2.3 Incremental lifetime cancer risk (ILCR) assessment

Assessment of the ILCR is an effective method to evaluate the degree of potentially adverse effects following exposure to pollutants in soils [21,23]. According to USEPA guidelines, the main exposure pathways are considered to be the accidental ingestion of soils, dermal contact with soils, and the inhalation of soil particles [10,21,22]. The ILCR values (unitless) for adults via different exposure pathways were calculated using the following formulas [21–23], as:

$$ILCR_{\mathrm{ingestion}}=\frac{C\times (CSF_{\mathrm{ingestion}}\times \sqrt[{3}]{(BW/70}))\times IR_{\mathrm{ingestion}}\times EF\times ED}{BW\times AT\times 10^{6}}$$ (9)

$$ILCR_{\mathrm{dermal}}=\frac{C\times (CSF_{\mathrm{dermal}}\times \sqrt[{3}]{(BW/70}))\times SA\times AF\times ABS\times EF\times ED}{BW\times AT\times 10^{6}}$$ (10)

$$ILCR_{\mathrm{inhalation}}=\frac{C\times (CSF_{\mathrm{inhalation}}\times \sqrt[{3}]{(BW/70}))\times IR_{\mathrm{inhalation}}\times EF\times ED}{BW\times AT\times PEF}$$ (11)

where ILCR_ingestion_, ILCR_dermal_ and ILCR_inhalation_ are the cancer risks via soil ingestion, dermal contact, and inhalation, respectively; C is the BaP_eq_ for the 16 PAHs compounds in soil samples (ng g^-1^) based on the TEFs (Table 2); EF is the exposure frequency (d y^-1^); IR_ingestion_ refers to the oral ingestion rate (mg d^-1^); IR_inhalation_ is the inhalation rate (m^3^ d^-1^); BW is the average bodyweight (kg); ED is the exposure duration (y); AT is the averaging time (d); SA is the surface skin area (cm^2^); ABS is the dermal absorption (unitless); AF is the relative skin adherence factor (mg cm^-2^); PEF is the soil dust production factor (m^3^ kg^-1^) and CSF_ingestion_, CSF_dermal_ and CSF_inhalation_ are carcinogenic slope factors for soil ingestion, dermal contact, and inhalation ((mg kg^-1^ d^-1^)^-1^), respectively. Here, the exposure parameters for adults were adopted from [22,25] and [26], which were shown in Table 3.

**Table 3:** Exposure parameters used in the incremental lifetime cancer risk (ILCR) assessment.

Parameters	Unit	Meaning	Value	Reference
BW	kg	Body weight	62	[22]
EF	d y^-1^	Exposure frequency	180	[22]
ED	y	Exposure duration	24	[22]
IR_inhalation_	m^3^ d^-1^	Inhalation rate	20	[25]
IR_ingestion_	mg d^-1^	Soil ingestion rate	100	[25]
SA	cm^2^	Surface area	5700	[25]
AF	mg cm^-2^	Adherence factor to skin	0.07	[25]
AT	d	Averaging time	25550	[22]
ABS	Unitless	Dermal absorption factor	0.13	[22]
PEF	m^3^ kg^-1^	Particle emission factor	1.36×10^9^	[22]
CSF_inhalation_	(mg kg^-1^ d^-1^)^-1^	Cancer slope factor via inhalation	3.85	[26]
CSF_ingestion_	(mg kg^-1^ d^-1^)^-1^	Cancer slope factor via ingestion	7.3	[26]
CSF_dermal_	(mg kg^-1^ d^-1^)^-1^	Cancer slope factor via dermal contact	25	[26]

Finally, the total ILCR value was the sum of the risks associated with different exposure pathways [21,23], as:

$$ILCR=ILCR_{\mathrm{ingestion}}+ILCR_{\mathrm{dermal}}+ILCR_{\mathrm{inhalation}}$$ (12)

### 2.4 Statistical analysis

In this study, PAH source identification and apportionment with PCA and MLR were performed using SPSS 18.0. The US EPA PMF model version 5.0 was also used to apportion the sources of PAHs and their toxicity. It is to be noted that the total PAH concentrations shown in this study were obtained from different papers. There exist some uncertainties with the use of deterministic values (such as the average concentrations) in evaluating contamination levels [42–44]. Fortunately, the Monte Carlo simulation with a Crystal Ball 7.2 software is a widely used probabilistic method that can take parameter uncertainties into account during risk prediction [43,44]. In this study, the best-fit distribution function of the PAH concentrations from different studies were first obtained with the assistance of different distribution functions, and the median values were used as PAH concentrations in their risk evaluation [42–46].

## 3 Results and discussion

### 3.1 PAH concentrations and distributions in different land use types

As shown in Table 1, PAH concentrations in different land use types showed large spatial variabilities with a range of 175.7–1989.0 ng g^-1^. PAH concentrations in surface soils can be divided into four contamination levels: not contaminated with PAHs (<200 ng g^-1^), weakly contaminated with PAHs (200–600 ng g^-1^), contaminated with PAHs (600–1000 ng g^-1^), and heavily contaminated with PAHs (>1000 ng g^-1^). Consequently, seven studies each in urban and suburban soils and six studies in rural soils were classified as contaminated and heavily contaminated (Table 1).

In urban soils, the total PAH concentrations were 360.0–1989.0 ng g^-1^ with the lowest PAH concentration observed in Nankai University in Tianjin and the highest PAH concentration observed in school grounds in Beijing [25,30]. All the measured total PAH concentrations in urban soils followed a pareto distribution with a median concentration of 789.7 ng g^-1^, indicating that soils were in the contaminated category (Table 1) on a large spatial scale.

In suburban soils, the lowest PAH concentration was observed in the in the Jinghai district (142.0 ng g^-1^) and the highest concentration in the Xiqing district (1490.0 ng g^-1^) of Beijing and Tianjin (See Table 1) [29]. Results from the Monte Carlo simulation suggested that their PAH concentrations followed a gamma distribution with a median concentration of 647.3 ng g^-1^ (classified as contaminated over a large spatial scale). The PAH concentrations in urban and suburban soils were similar to those in surface soils in Jena, Germany (211–2048 ng g^-1^ with a median concentration of 677 ng g^-1^), which were higher than those in surface soils in Caserta, Italy (10.0–4191 ng g^-1^ with a mean concentration of 137±524 ng g^-1^). However, the concentrations were much lower than those in Lisbon, Portugal (6.3–22700 ng g^-1^ with a mean concentration of 1540 ng g^-1^) and Ahvaz, Iran (75.8–15508.0 ng g^-1^, with a mean concentration of 1732.8 ng g^-1^) [4–6,47]. It is noteworthy that the median PAH concentrations in suburban soils were much lower than in urban soils, which may be attributed to the higher emission rates from intensive human activities in urban areas [23,29].

In rural soils, the highest PAH concentrations were observed in agricultural and gardening soils in Tianjin (1258.6–1295.8 ng g^-1^), while the lowest PAH concentration was observed in agricultural facility areas in Tianjin (175.7 ng g^-1^) (See Table 1) [12,13,34]. Probabilistic results from the Monte Carlo simulation suggested that all the PAH concentrations followed a log-normal distribution with a median concentration of 390.8 ng g^-1^ (classified as weakly contaminated over a large spatial scale), which was higher than that in surface soils in rural areas of southern Italy (1.87–11353 ng g^-1^ with a mean concentration of 333.3 ng g^-1^) and Lakki Marwai, Pakistan (222 ng g^-1^), but was significantly lower than that in Dhanbad, India (1019–10856 ng g^-1^ with a mean of 3488 ng g^-1^), and Kumasi Metropolis, Ghana (1398 ng g^-1^) [2,3,8,35].

### 3.2 PAH source identification and toxicity apportionment

#### 3.2.1 Source identification with diagnostic ratios and PCA

**Fig 1:**
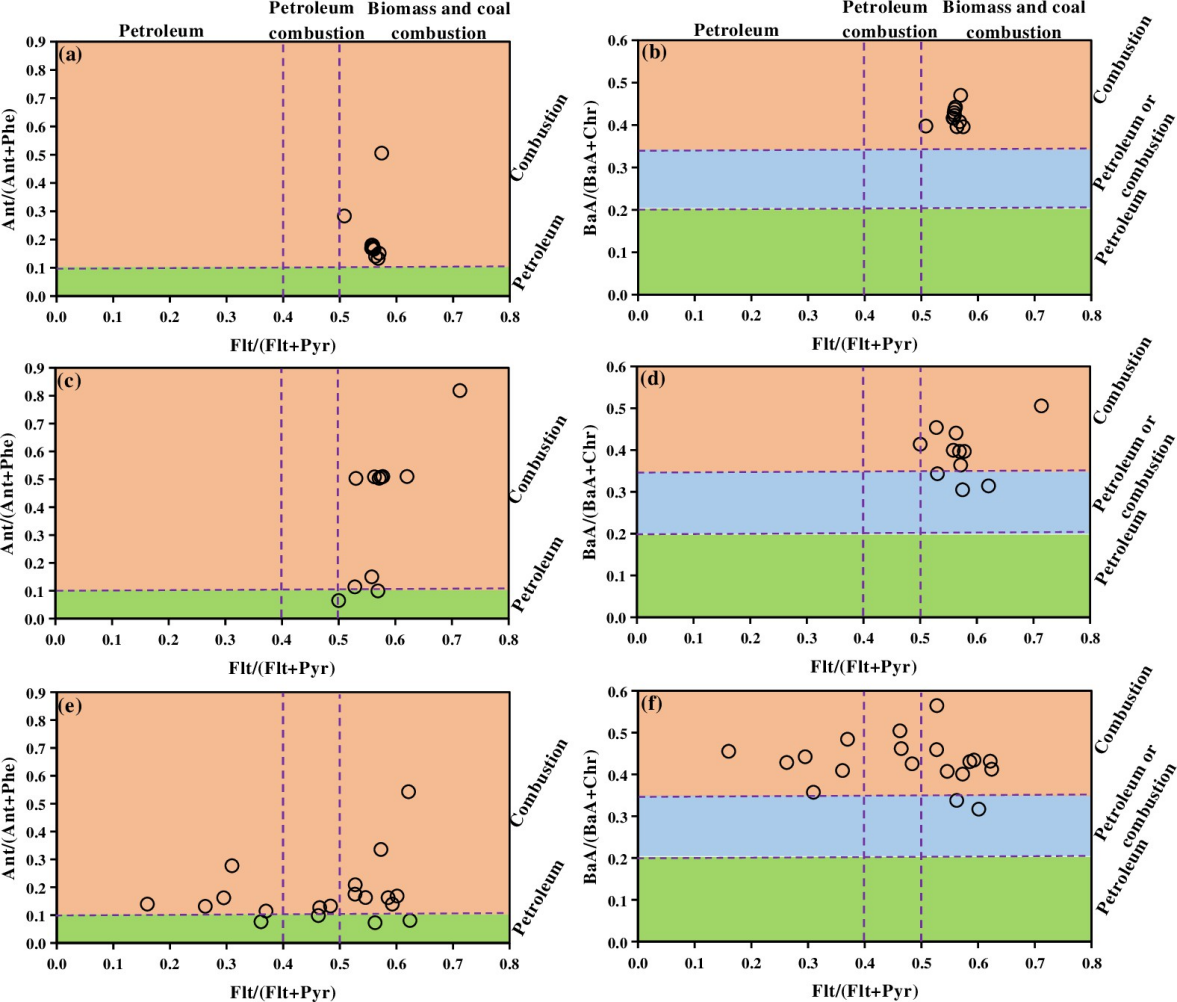
Cross plots for PAH isomeric ratios in surface soils in urban (a, b), suburban (c, d) and rural area (e, f). The calculations of source ratios followed the methods described by Yunker et al [15].

The three most frequently used molecular diagnostic ratios, i.e., BaA/(BaA+Chr), Flt/(Flt+Pyr), and Ant/(Ant+Phe), were investigated in this study, and their cross plots are shown in Fig 1. In urban and suburban soils, the values of these three diagnostic ratios were similar to BaA/(BaA+Chr)>0.35, Flt/(Flt+Pyr)>0.5, and Ant/(Ant+Phe)>0.1, suggesting that wood and coal combustion were the dominant sources. The ratios of BaA/(BaA+Chr) and Ant/(Ant+Phe) for the PAHs in rural soils were also >0.35 and >0.1, respectively, while the ratio of Flt/(Flt+Pyr) varied within the range of 0.15–0.75, which suggests a mixed source of petroleum and combustion.

We also identified the PAH sources in different land use types using a PCA model. The rotated loadings for different extracted PCs are listed in Table 4. In urban soils, two PCs were extracted (PC1–2), accounting for 96.5% of the total variance. PC1 accounted for 54.9% of the total variance, which was predominantly due to Phe, Ant, Flt, Pyr, BaA, Chr, BbF, BkF, BaP, IND, DBahA, and BghiP. The profile of Phe, Ant, Flt, and Pyr from biomass and coal combustion has been reported in previous studies [1,17]. Pyr, BkF, BbF, BaP, and DBahA have been considered as tracers of vehicle exhaust emissions [1,7,48]. Additionally, high relative abundances of IND, DBahA, and BghiP have also been frequently observed in diesel and gas engine emissions [1,48]. Thus, PC1 was a mixed source of biomass and coal combustion and vehicular emissions. PC2 comprised 41.53% of the total variance with high loadings on Naph, Acy, Ace, Flu, Phe, Ant, Flt, Pyr, Chr, and BbF, and moderate loadings on BaP and BaA. The dominance of low molecular weight (LMW) PAHs (for example Naph, Acy, Ace, Flu, Ant, and Phe) suggests a petrogenic source [7,17,49]. Furthermore, the profiles of Phe, Ant, Flt, and Pyr are considered as tracers of biomass and coal combustion [17,49]. Therefore, PC2 represents a mixed petrogenic and biomass and coal combustion source.

**Table 4:** The total variance explained and component matrix of PAHs in different land use types in Beijing and Tianjin.

PAHs	Urban soils		Suburban soils			Rural soils			
	PC1	PC2	PC1	PC2	PC3	PC1	PC2	PC3	PC4
Naph	-0.10	**0.94**	-0.14	**0.93**	-0.23	0.15	0.16	0.06	**0.79**
Acy	0.37	**0.89**	-0.20	-0.17	**0.95**	**0.89**	0.25	-0.20	-0.07
Ace	0.26	**0.92**	-0.19	-0.20	**0.94**	0.12	**0.96**	0.02	0.21
Flu	0.40	**0.87**	-0.16	**0.85**	0.12	-0.06	-0.00	**0.94**	0.14
Phe	**0.68**	**0.71**	0.09	**0.96**	0.09	**0.66**	0.02	-0.03	**0.74**
Ant	**0.53**	**0.79**	-0.05	0.24	**0.94**	-0.14	-0.03	0.15	**0.83**
Flt	**0.77**	**0.62**	**0.51**	**0.83**	-0.18	**0.97**	-0.04	-0.14	0.17
Pyr	**0.75**	**0.64**	**0.59**	**0.75**	-0.23	**0.99**	-0.02	0.05	0.11
BaA	**0.88**	0.47	**0.76**	**0.59**	0.04	**0.84**	0.43	0.18	0.13
Chr	**0.80**	**0.58**	**0.63**	**0.74**	-0.11	**0.90**	0.12	0.29	0.22
BbF	**0.69**	**0.69**	**0.91**	-0.00	-0.34	**0.76**	0.24	**0.58**	0.02
BkF	**0.97**	0.17	0.34	-0.14	-0.57	0.31	0.19	**0.88**	0.07
BaP	**0.91**	0.41	**0.85**	0.42	-0.11	**0.84**	0.39	0.32	0.01
IND	**0.98**	0.16	**0.96**	-0.17	-0.13	0.29	**0.93**	0.16	-0.06
DBahA	**0.98**	0.11	0.03	-0.23	**0.96**	**0.92**	0.17	0.20	-0.02
BghiP	**0.94**	0.32	**0.99**	0.00	-0.11	**0.86**	0.41	0.26	-0.03
Eigenvalues	13.04	2.40	7.79	3.96	3.02	9.24	2.22	1.92	1.57
Variance	54.98%	41.53%	33.47%	32.03%	26.83%	49.02%	15.95%	15.47%	13.06%
Cumulative variance	54.98%	96.52%	33.47%	65.50%	92.32%	49.02%	64.97%	80.44%	93.50%
Contributions	0.62	0.38	0.43	0.32	0.25	0.46	0.14	0.13	0.26

Factor loading ≥ 0.50 are in bold.

In suburban soils, three principal components (PC1–3) were obtained, which account for 92.3% of the total variance. PC1 contributed 33.5% of the total variance, among which BbF, BaP, IND, and BghiP possessed relatively high factor loadings (>0.80) and Flt, Pyr, BaA, Chr, and BkF possessed moderate factor loadings (0.30–0.80). High loadings of BaP, IND, and BghiP suggested the PAH emissions originated from vehicular emissions [8,48], whereas moderate loadings for Flt, BaA, Pyr, Chr, and BkF represent the PAH emission from biomass and coal combustion [1,17]. Therefore, PC1 represents a mixed source of biomass and coal combustion and vehicular emissions. PC2 accounted for 32.0% of the total variance with high loadings for Naph, Flu, Phe, and Flt and moderate loadings for Pyr, BaA, and Chr. These loadings suggested a mixed emission source of petrogenic and biomass and coal combustion [7,17]. PC3 accounted for 26.8% of the total variance, which was featured by the high loadings for Ace, Acy, Ant, and DBahA. The dominance of LMW PAHs (for example the Ace, Acy, and Ant) suggested a petrogenic source [17,49], whereas the high loading on DBahA suggested a vehicular emission source [7,8]. Therefore, PC3 represents a mixed petrogenic and vehicular emissions source.

In rural soils, four principal components (PCs) were extracted (PC1–4), which accounted for 93.5% of the total variance. PC1 can explain 49.0% of the total variance with high loadings on Acy, Phe, Flt, BbF, DBahA, Pyr, BaP, BaA, Chr, and BghiP. Their loadings suggested a mixed source of biomass and coal combustion and vehicular emissions [1,8,17]. PC2 was responsible for 15.9% of the total variance, which was featured by the high loading for Ace and moderate loadings for BbF and BkF. Their PAH profiles suggested a mixed source of petrogenic and vehicle emissions [7,8,48]. PC3 and PC4 accounted for 15.5% and 13.1% of the total variance with high loadings for Phe, BbF, and BkF for PC3, and Naph, Phe, and Ant for PC4, respectively, suggesting a PAH source from biomass and coal combustion and petrogenic sources [8,17,48].

Consequently, the PAHs in different land use types share similar PAH sources, i.e., biomass and coal combustion, petrogenic source, and vehicular emission. In order to determine percentage contributions of PAHs from different sources in different land use types, an MLR model was also used in this study. The results are shown in Table 4 and the contributions for each PC varies in the range of 13%–62%.

#### 3.2.2 Source identification and toxicity apportionment with a PMF-TEQ method

**Fig 2:**
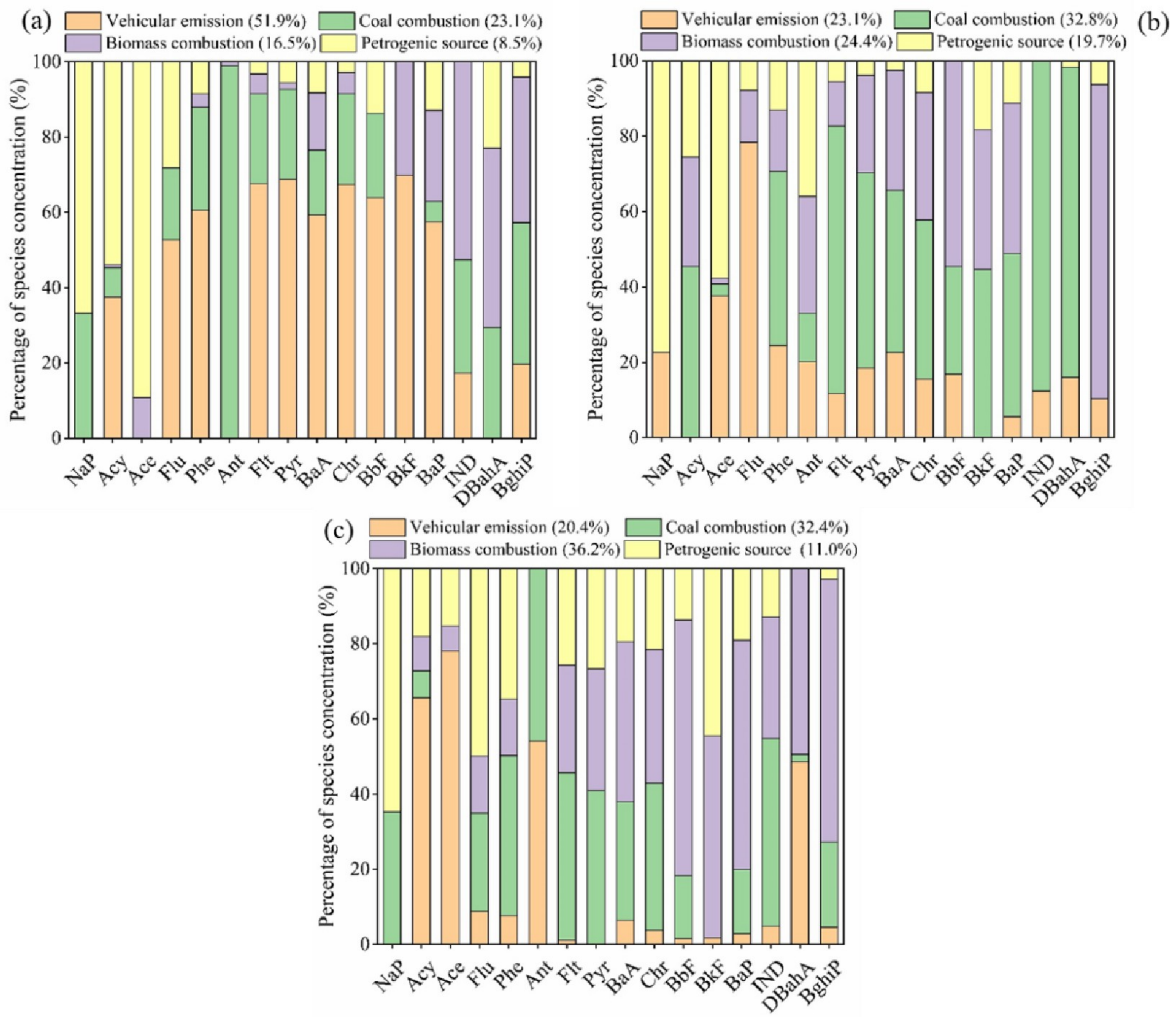
Source profiles of each PMF factor for PAHs in urban soils (a), suburban soils (b) and rural soils (c).

In order to identify the most appropriate factors for the data, a different number of factors, ranging from three to eight, was initially explored with the PMF model. The four-factor model was by far the most appropriate for all land use types, with *R*^*2*^ values ranging from 0.91 to 1.00, and was therefore selected for further analysis. In this study, four main sources were identified using the PMF model, including vehicular emissions, coal combustion, biomass combustion, and petrogenic sources. The source profiles of each PMF factor for PAHs in different land use types is shown in Fig 2.

In urban soils, factor 1 accounted for 51.9% of the total measured PAHs, which was dominated by Flt, Pyr, BaA, Chr, BbF, BkF, and BghiP. The predominance of high molecular weight (HMW) PAHs suggested a vehicular emission source [7,8]. Factor 2 was responsible for 23.1% of the total PAHs. It was dominated by Ant, Flt, Pyr, BbF, and BkF, which are considered as tracers of coal combustion [1,17]. Factor 3 was responsible 16.5% of the total PAHs with high loadings for BkF, BaP, IND, DBahA, and BghiP, which are considered to be tracers of biomass combustion [17,49]. Factor 4 accounted for 8.5% of the total PAHs with high loading on LMW PAHs such as Naph, Acy, Ace, and Flu, suggesting a petrogenic source [7,17,49].

With the tracers presented in [1,7,17], and [49], different PAH sources and their associated contributions in suburban and rural soils were also identified and quantified. As a result, the calculated contributions of vehicular emissions, coal and biomass combustion, and petrogenic source to PAH concentrations in suburban and rural soils were 23.1%, 32.8%, 24.4%, 19.7% and 20.4%, 32.4%, 36.2%, and 11.0%.

In all three land use types, the PAH sources obtained from the PMF model were consistent with the results from the PCA model, thereby verifying the accuracy of our results. Furthermore, the BaP_TEQ_ values for the 16 PAHs were also calculated based on their TEF values in order to evaluate their toxicity. The BaP_TEQ_ values determined using all 16 PAH compounds in urban, suburban, and rural soils were 121.6 ng g^-1^, 72.8 ng g^-1^, and 48.6 ng g^-1^, respectively. The highest BaP_TEQ_ values were observed in urban soils, likely due to their high PAH concentrations and the contributions of HMW PAH compounds with high TEF values. Compared with other studies, the BaP_TEQ_ values in urban soils were even higher than that of surface soils in an industrialized area in Dilovasi (which varied from 1.7 ng g^-1^ to 1167.9 ng g^-1^ with a mean value of 100.8±164.66 ng g^-1^) and the urban soils in Lebanon (38.4±21.7 ng g^-1^), but was much lower than that of the urban soil in Dhanbad, India (720 ng g^-1^) [7,8,39].

**Fig 3:**
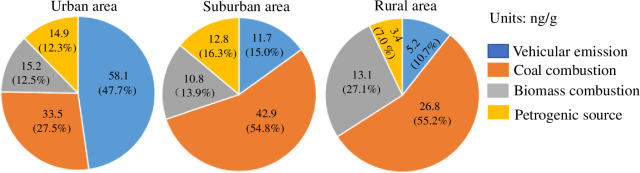
Source contributions to BaP_TEQ_ for PAHs in urban soils (a), suburban soils (b) and rural soils (c).

Fig 3 shows the source contributions to BaP_TEQ_ for the 16 PAH compounds in different land use types. In urban soils, vehicular emission (47.7%) and coal combustion (27.5%) contributed the most to carcinogenic risks, followed by biomass combustion (12.5%) and petrogenic sources (12.3%). In suburban soils, coal combustion contributed the most to the BaP_TEQ_ concentrations, while the final three sources have approximately equal contributions (13.9%–16.3%). In rural soils, the contributions of coal and biomass combustion were much higher than vehicular emissions and petrogenic sources, and presented the main carcinogenic risk. Thus, the vehicular emissions and coal combustion contributed most to the BaP_TEQ_ concentrations in all three land use types.

### 3.3 Incremental lifetime cancer risk (ILCR) assessment

In order to quantify the cancer risk due to PAH exposure in different land use types within Beijing and Tianjin, an ILCR assessment model proposed by USEPA guidelines which accounts for different exposure pathways (i.e., soil ingestion, dermal contact and air inhalation) was used in this study [10,21–23,50]. Studies suggested that, although children are more sensitive to pollutants, the soil ingestion rate and exposure duration for adults and teens are greater. The cancer risks for the three different age groups are adults > teens > children [46]. In this study, we only discussed the cancer risks for adults.

With the BaP_TEQ_ concentrations in different land use types and the exposure parameters shown in Table 3, we evaluated the ILCR for adults [25,26,50]. Considering the uncertainties in the calculation of BaP_TEQ_ concentration from large spatial scales, we evaluated their cancer risks using the Monte Carlo simulation. The distributions patterns of the ILCR were first obtained, and the 95^th^ percentile values were used as high-end estimates in risk prediction [21–23,50].

**Fig 4:**
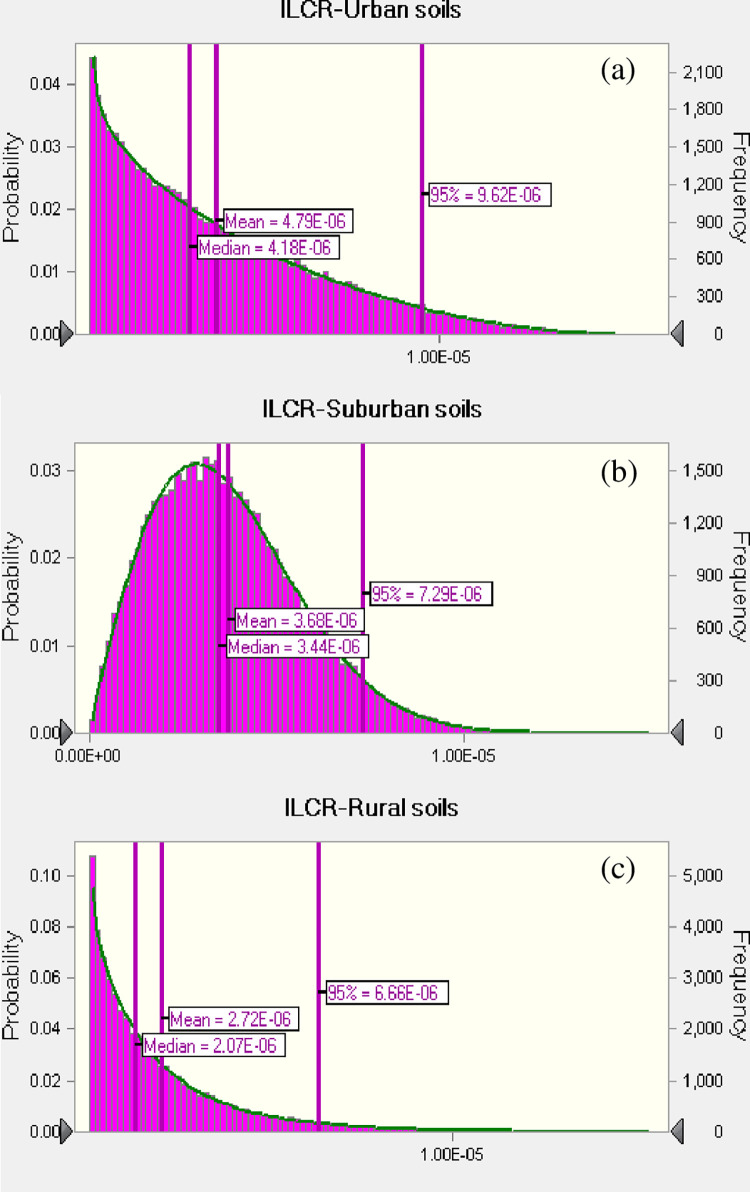
Predicted probability density functions of incremental lifetime cancer risk (ILCR) for adults exposed to the PAHs in urban soils (a), suburban soils (b) and rural soils (c).

As shown in Fig 4, the 95^th^ of ILCR values were estimated to be 9.6×10^−6^ (pareto distribution), 7.3×10^−6^ (log-normal distribution), and 6.7×10^−6^ (pareto distribution) for adults exposed to the soil PAHs in urban, suburban, and rural soils, respectively. As suggested by the USEPA, the cancer risks can be classified into three categories. ILCRs lower than 10^−6^ are considered as the safe level, between 10^−6^ and 10^−4^ indicates a low risk level, and ILCRs higher than 10^−4^ indicate marginal safety [10,11]. The ILCRs for adults exposed to PAHs in all three land use types were higher than 10^−6^, but lower than 10^−4^, which indicates a low risk level. Regarding different exposure pathways, dermal contact and soil ingestion represented major cancer risks, whereas the contribution of inhalation was minor (not shown in this study). This is similar to the results reported in [51] and [52].

### 3.4 Limitation

Uncertainties are inherent in source identification, toxicity apportionment and the health risk assessment, which stems from a lack of knowledge about their emission sources and the factors affecting exposure or toxicity assessment [45,46]. More receptor models, for example the CMB model, should be used to verify our modeled result. In addition, more exposure pathways, for example PAH intake via food ingestion, should be considered in the risk assessment. And more accurate exposure parameters, dose-response data on carcinogenicity and TEF values should be obtained in the future [45,46].

## 4. Conclusions

We reported the pollution statues of PAHs in different land use types in Beijing and Tianjin. The total PAH concentration in urban soils was the highest, followed by suburban soils and finally rural soils. Diagnostic ratios suggested that the PAHs in urban soils and suburban soils mainly originated from wood and coal combustion, whereas those in rural soils originated from a mixed source of petroleum and combustion. The PCA model source identification displayed different PAH emission sources, including biomass and coal combustion, vehicular emissions, and petrogenic processes, which were identified with contributions ranging from 13% to 62%. The PMF model showed that the vehicular emissions and coal combustion in urban soils and the vehicular emissions, coal combustion, and biomass combustion in suburban and rural soils dominated the total PAH concentrations (>85%), which was consistent with the results from the PCA model. Toxicity apportionment analysis suggested that vehicular emission and coal combustion contributed the most to the BaP_TEQ_ concentrations, therefore dominating the carcinogenic potencies in all three land use types. Results from the ILCR model suggested that the cancer risks for adults varied in the range of 10^−6^–10^−4^, which indicates a low risk level.

## Supporting information

S1 Table Summary of quality control and instrumental analysis in different studies. (DOCX)
