# Supplementary material for: Source identification and toxicity apportionment of polycyclic aromatic hydrocarbons in surface soils in Beijing and Tianjin using a PMF-TEQ method
Source: PLoS One. 2022 Jun 30;17(6):e0268615. doi: 10.1371/journal.pone.0268615 (PMC9246166; doi:10.1371/journal.pone.0268615)
Supplement: S1 Table — (DOCX) [file pone.0268615.s001.docx]

**Supply material for** **“Source identification and toxicity apportionment of polycyclic aromatic hydrocarbons in surface soils in Beijing and Tianjin using a PMF-TEQ method”**

**Huashuang Zhang^1‡^, Qi Huang^2‡^, Ping Han^3^, Zhicheng Zhang^2^, Shengtao Jiang^2^*, Wei Yang^4^***

^1^ Institute for Brain Research and Rehabilitation, South China Normal University, Guangzhou, Guangdong, China

^2^ College of Life Science, Taizhou University, Taizhou, Zhejiang, China

^3^ Shandong Urban Construction Vocational College, Jinan, Shandong, China

**^4^** Polar and Marine Research Institute, Jimei University, Xiamen, Fujian, China

*Correspondence author

Email: [jst80@126.com](mailto:jst80@126.com) (SJ)

Email: [1061713103@qq.com](mailto:1061713103@qq.com) (WY)

^‡^ HZ and QH are co-first authors.

**Table S1. Summary of quality control and instrumental analysis in different studies.**

| Land use types | Soil descriptions | Total PAHs  (ng g^-1^) | BaPeq  (ng g^-1^) | Recovery | Quality control | Method Detection  Limit (ng g^-1^) | Measurements | Reference |
| --- | --- | --- | --- | --- | --- | --- | --- | --- |
| Urban  Soils | Surface soil in urban parks in Beijing | 460.0 | 50.7 | 58-127% | Blank samples, parallel samples, and certified reference PAHs | -- | GC-MS | [1] |
|  | Urban soils in Beijing | 1228.0 | 159.2 | 64-119% for 16 PAHs | Blank samples, parallel samples, and certified reference PAHs | -- | GC-MS | [2] |
|  | Surface soils in Nankai University, Tianjin | 360.0 | 36.9 | 67.2-122% for 16 PAHs | Blank samples, parallel samples, and certified reference PAHs | 1.2-8.3 | GC-MS | [3] |
|  | Surface soils in Beijing | 1082.6 | 180.7 | 61.75-102.84% for 16 PAHs | Matrix spike, laboratory control (method blank) and duplicate unspiked samples | 0.02-3.12 | GC-MS | [4] |
|  | Surface soil in schools in Beijing | 1989.0 | 286.6 | 64% for Naph and 86-119% for the remaining 15 PAHs | Replicated analyses | -- | GC-MS | [5] |
|  | Surface soil in parks in Beijing | 1285.0 | 170.6 | 64% for Naph and 86-119% for the remaining 15 PAHs | Replicated analyses | -- | GC-MS | [5] |
|  | Surface soil in roadside with heavy traffic in Beijing | 1026.0 | 135.0 | 64% for Naph and 86-119% for the remaining 15 PAHs | Replicated analyses | -- | GC-MS | [5] |
|  | Surface soil in residential area in Beijing | 811.0 | 98.7 | 64% for Naph and 86-119% for the remaining 15 PAHs | Replicated analyses | -- | GC-MS | [5] |
|  | Surface soil in plantation area in Beijing | 673.0 | 85.3 | 64% for Naph and 86-119% for the remaining 15 PAHs | Replicated analyses | -- | GC-MS | [5] |
|  | Surface soil in roadside with light traffic in Beijing | 538.0 | 68.9 | 64% for Naph and 86-119% for the remaining 15 PAHs | Replicated analyses | -- | GC-MS | [5] |
|  | Surface soil in vacant area in Beijing | 523.0 | 65.4 | 64% for Naph and 86-119% for the remaining 15 PAHs | Replicated analyses | -- | GC-MS | [5] |
| Suburban  Soils | Surface soils in Xiqing, Tianjin | 1490.0 | 178.7 | 67.2-122% for 16 PAHs | Blank samples, parallel samples, and certified reference PAHs | 1.2-8.3 | GC-MS | [3] |
|  | Surface soils in Jinnan, Tianjin | 708.0 | 85.4 | 67.2-122% for 16 PAHs | Blank samples, parallel samples, and certified reference PAHs | 1.2-8.3 | GC-MS | [3] |
|  | Surface soils in Beicheng, Tianjin | 904.0 | 69.6 | 67.2-122% for 16 PAHs | Blank samples, parallel samples, and certified reference PAHs | 1.2-8.3 | GC-MS | [3] |
|  | Surface soils in Dongli, Tianjin | 699.0 | 48.8 | 67.2-122% for 16 PAHs | Blank samples, parallel samples, and certified reference PAHs | 1.2-8.3 | GC-MS | [3] |
|  | Surface soils in Jinghai, Tianjin | 142.0 | 12.9 | 67.2-122% for 16 PAHs | Blank samples, parallel samples, and certified reference PAHs | 1.2-8.3 | GC-MS | [3] |
|  | Surface soils in Jixian, Tianjin | 382.0 | 32.7 | 67.2-122% for 16 PAHs | Blank samples, parallel samples, and certified reference PAHs | 1.2-8.3 | GC-MS | [3] |
|  | Surface soils in Xiqing, Tianjing | 422.8 | 57.1 | 63.1-71.0-97.8% for 16 PAHs | Blank samples, parallel samples, and certified reference PAHs | 36531.0 | HPLC | [6] |
|  | Surface soils from Tianjin coastal new region | 932.0 | 124.2 | 70.4-117% for 16 PAHs | Laboratory blanks, matrix-spiked recoveries, and duplicates | 0.03-0.46 | GC-MS | [7] |
|  | Surface soil in Tongzhou District, Beijing | 1004.1 | 158.4 | 76.0-126% for 16 PAHs | Replicated analyses | 0.02-4.88 | GC-MS | [8] |
|  | Surface soils in suburban area of Beijing | 321.8 | 38.1 | 51-95% for 16 PAHs | Replicated analyses | 0.05-0.39 | GC-MS | [9] |
|  | Surface soils in suburban area of Beijing and Tianjin | 622.4 | 54.6 | 66-114% for 16 PAHs | Laboratory blanks, matrix-spiked recoveries, and duplicates | 0.29-1.02 | GC-MS | [10] |
| Rural  Soils | Agricultural soil in suburb of Beijing | 460.8 | 24.8 | 75-108% for 16 PAHs | Laboratory blanks, matrix-spiked recoveries, and duplicates | -- | GC-MS | [11] |
|  | Surface soils in rural area of Beijing | 219.2 | 27.3 | 51-95% for 16 PAHs | Laboratory blanks, matrix-spiked recoveries, and duplicates | 0.05-0.39 | GC-MS | [9] |
|  | Surface soils in rural area of Beijing and Tianjin | 195.3 | 14.8 | 66-114% for 16 PAHs | Laboratory blanks, matrix-spiked recoveries, and duplicates | 0.29-1.02 | GC-MS | [10] |
|  | Arable soils of Beijing | 489.6 | 71.0 | 52.1-124.1% for 16 PAHs | Laboratory blanks, matrix-spiked recoveries, and duplicates | 0.1-7.2 | GC-MS | [12] |
|  | Agricultural soil in Tianjin | 1295.8 | 185.6 | 64.6-111.9% for 16 PAHs | Replicated analyses | 0.16-9.36 | GC-MS | [13] |
|  | Surface soil from garden in Tianjin | 1258.6 | 126.8 | 63.5-104.7% for Naph-d8, Ace-d10, Chr-d12, Per-d12 and Phe-d10 | Replicated analyses | 0.03-0.37 | GC-MS | [14] |
|  | Surface soil from cropland in Tianjin | 624.7 | 114.4 | 63.5-104.7% for Naph-d8, Ace-d10, Chr-d12, Per-d12 and Phe-d10 | Replicated analyses | 0.03-0.37 | GC-MS | [14] |
|  | Surface soil from dryland in Tianjin | 1003.9 | 97.1 | 63.5-104.7% for Naph-d8, Ace-d10, Chr-d12, Per-d12 and Phe-d10 | Replicated analyses | 0.03-0.37 | GC-MS | [14] |
|  | Surface soil in residential areas of Tianjin | 481.8 | 4.5 | 80.5-114.6% | Laboratory blanks, matrix-spiked recoveries, and duplicates | 0.01-1.9 | HPLC | [15] |
|  | Surface soil in residential areas of Tianjin | 435.1 | 10.8 | 80.5-114.6% | Laboratory blanks, matrix-spiked recoveries, and duplicates | 0.01-1.9 | HPLC | [15] |
|  | Surface soil in residential areas of Tianjin | 289.1 | 3.7 | 80.5-114.6% | Laboratory blanks, matrix-spiked recoveries, and duplicates | 0.01-1.9 | HPLC | [15] |
|  | Surface soil in agricultural facility areas of Tianjin | 175.7 | 6.9 | 80.5-114.6% | Laboratory blanks, matrix-spiked recoveries, and duplicates | 0.01-1.9 | HPLC | [15] |
|  | Surface soil in agricultural facility areas of Tianjin | 296.1 | 25.5 | 80.5-114.6% | Laboratory blanks, matrix-spiked recoveries, and duplicates | 0.01-1.9 | HPLC | [15] |
|  | Surface soil in agricultural facility areas of Tianjin | 229.3 | 11.9 | 80.5-114.6% | Laboratory blanks, matrix-spiked recoveries, and duplicates | 0.01-1.9 | HPLC | [15] |
|  | Surface soil in agricultural facility areas of Tianjin | 286.0 | 17.0 | 80.5-114.6% | Laboratory blanks, matrix-spiked recoveries, and duplicates | 0.01-1.9 | HPLC | [15] |
|  | Surface soil in farmland around livestock breeding areas of Tianjin | 772.9 | 10.9 | 80.5-114.6% | Laboratory blanks, matrix-spiked recoveries, and duplicates | 0.01-1.9 | HPLC | [15] |
|  | Surface soil in farmland around livestock breeding areas of Tianjin | 259.9 | 4.1 | 80.5-114.6% | Laboratory blanks, matrix-spiked recoveries, and duplicates | 0.01-1.9 | HPLC | [15] |
|  | Surface soil in farmland around industrial areas of Tianjin | 323.3 | 55.2 | 80.5-114.6% | Laboratory blanks, matrix-spiked recoveries, and duplicates | 0.01-1.9 | HPLC | [15] |
|  | Vegetable soils from the Beijing-Tianjin | 602.5 | 111.4 | 86.5% for Phe-d10 and 67.9-104.5% for individual PAHs | Method blanks (solvent), duplicate samples and spiked blanks | 1.5-3.6 | GC-MS | [16] |

**Reference**

1. Qu Y, Gong Y, Ma J, Wei H, Liu Q, Liu L, et al. Potential sources, influencing factors, and health risks of polycyclic aromatic hydrocarbons (PAHs) in the surface soil of urban parks in Beijing, China. Environ Pollut. 2020;260: 114016.

2. Peng C, Chen W, Liao X, Wang M, Ouyang Z, Jiao W, et al. Polycyclic aromatic hydrocarbons in urban soils of Beijing: Status, sources, distribution and potential risk. Environ Pollut. 2011;159: 802-808.

3. Zhu Y, Tian J, Wei E, Wei F. Characteristics，sources apportionment and ecological risks assessment of polycyclic aromatic hydrocarbons in soils of Tianjin, China. Environ Chem. 2014;33: 248-255. (in Chinese)

4. Liu S, Xia X, Yang L, Shen M, Liu R. Polycyclic aromatic hydrocarbons in urban soils of different land uses in Beijing, China: Distribution, sources and their correlation with the city’s urbanization history. J Hazard. Mater. 2010;177: 1085-1092.

5. Peng C, Ouyang Z, Wang M, Chen W, Jiao W. Vegetative cover and PAHs accumulation in soils of urban green space. Environ Pollut. 2012;161: 36-42.

6. Wang D, Luo M, Zhang Q, Dai H, Liu Y, Wang L, et al. Distribution Characteristics of Polycyclic Aromatic Hydrocarbons in Different Functional Zones of Soils from Xiqing District in Tianjin, China. J Agro-Environ Sci. 2012;31: 2374-2380. (in Chinese)

7. Shi R, Li X, Yang Y, Fan Y, Zhao Z. Contamination and human health risks of polycyclic aromatic hydrocarbons in surface soils from Tianjin coastal new region, China. Environ Pollut. 2021;268: 115938.

8. An Y, Huang Y, Sun C, Li D, Huang D. Source apportionment and risk assessment of PAHs in soil from a renewal area in the Tongzhou District of Beijing. Hydro Eng Geo. 2017;44: 112-120. (in Chinese)

9. Peng C, Wang M, Zhao Y, Chen W. Distribution and risks of polycyclic aromatic hydrocarbons in suburban and rural soils of Beijing with various land uses. Environ Monit and Assess. 2016;188: 162.

10. Wang W, Simonich S, Xue M, Zhao J, Zhang N, Wang R, et al. Concentrations, sources and spatial distribution of polycyclic aromatic hydrocarbons in soils from Beijing, Tianjin and surrounding areas, North China. Environ Pollut. 2010;158: 1245-1251.

11. Zhou J, Zhang J, Liu X, Feng Y, Wang H, Xu S, et al. Pollution characteristics and risk assessment of PAHs in agricultural soil in suburb of Beijing. J Agricul Res Environ. 2019;36: 534-540.

12. Liu H, Yu X, Liu Z, Sun Y. Occurrence, characteristics and sources of polycyclic aromatic hydrocarbons in arable soils of Beijing, China. Ecotoxicol Environ Saf. 2018;1159: 120-126.

13. Chen R, Lv J, Zhang W, Liu S, Feng J. Polycyclic aromatic hydrocarbon (PAH) pollution in agricultural soil in Tianjin, China: a spatio-temporal comparison study. Environ Earth Sci. 2015;74: 2743-2748.

14. Shi R, Xu M, Liu A, Tian Y, Zhao Z. Characteristics of PAHs in farmland soil and rainfall runoff in Tianjin, China. Environ Monit and Assess. 2017;189: 558.

15. Xu Y, Dai S, Meng K, Wang Y, Ren W, Zhao L, et al. Occurrence and risk assessment of potentially toxic elements and typical organic pollutants in contaminated rural soils. Sci Total Environ. 2018;630: 618-629.

16. Wang Y, Qiao M, Liu Y, Arp H, Zhu Y. Comparison of polycyclic aromatic hydrocarbon uptake pathways and risk assessment of vegetables from waste-water irrigated areas in northern China. J Environ Monitor. 2011;13: 433-439.
